# Immunohistochemical TTF-1 and Napsin a Expression in Gastrointestinal Adenocarcinomas—Low Frequency but an Important Pitfall

**DOI:** 10.3390/diagnostics15121490

**Published:** 2025-06-11

**Authors:** Petar Noack, Claudia Grosse, Simon Eschemann, Bastian Dislich, Rupert Langer

**Affiliations:** 1 Department of Pathology and Molecular Pathology, Kepler University Hospital, Johannes Kepler University Linz, Med Campus III, Krankenhausstraße 9, 4021 Linz, Austria; petar.noack@kepleruniklinikum.at (P.N.); claudia.grosse@kepleruniklinikum.at (C.G.); 2 Medical Faculty, Johannes Kepler University Linz, Altenberger Straße 69, 4040 Linz, Austria; simoneschemann@gmail.com; 3 Institute of Tissue Medicine and Pathology, University of Bern, Murtenstraße 31, 3010 Bern, Switzerland; bastian.dislich@unibe.ch

**Keywords:** colon cancer, gastric cancer, esophageal cancer, TTF-1, Napsin A, TMA

## Abstract

**Background/Objectives**: TTF-1 and Napsin A are immunohistochemical markers that are widely used for the diagnosis of lung adenocarcinomas or thyroid carcinomas, as well as the characterization of metastases. However, several publications have reported the aberrant expression of one or both markers in extrathoracic malignancies, including gastrointestinal adenocarcinomas. The goal of our study was to determine the frequency of TTF-1- and Napsin A-positive neoplasms in cohorts consisting of esophageal, gastric and colorectal adenocarcinomas. **Methods**: Buffered formalin-fixed paraffin-embedded tumor tissues from 854 patients with primary resected gastrointestinal and esophageal carcinomas were placed in tissue microarrays (TMAs) for investigation. Between two and six tumor cores were analyzed for each case. For immunohistochemical staining, we used TTF-1 (SPT24 clone) and Napsin A (IP64 clone). Tumors were considered positive if at least 5% of their tumor cells showed weak nuclear (TTF-1) or cytoplasmic (Napsin A) staining. **Results**: In total, 16 cases showed positive staining for TTF-1, alongside 7 cases for Napsin A. The greatest proportion of TTF-1- and/or Napsin A-positive tumors was found among esophageal adenocarcinomas (5/125 cases; 4%). Co-expression of TTF-1 and Napsin A was found in five cases, including three esophageal and two gastric adenocarcinomas. In colorectal carcinomas, co-expression of these markers was not detected. **Conclusions**: TTF-1 and Napsin A are useful immunohistochemical makers for establishing the diagnosis of pulmonary adenocarcinoma. Additionally, knowing that a proportion of gastrointestinal neoplasms express these markers can help to avoid diagnostic misinterpretations.

## 1. Introduction

Thyroid transcription factor-1 (TTF-1), a homeodomain-containing DNA-binding protein comprising 371 amino acids with a molecular weight of 38 kDa, belonging to the NKX2 gene family located on chromosome 14q13, was originally identified in epithelial thyroid follicular cells, but subsequently also type II pneumocytes and Clara cells of the lung [1,2,3,4,5,6,7]. In tissue analysis, immunohistochemistry (IHC) using anti-TTF-1 antibodies reveals nuclear staining in these cell types, as well as in corresponding neoplastic cells. Anti-TTF-1 antibodies are commonly used in routine pathology diagnostics, assisting in the differential diagnosis between squamous cell carcinomas and adenocarcinomas of the lung (which usually show TTF-1 positivity). They also help to differentiate between adenocarcinomas of the lung and the thyroid, where this immunohistochemical marker is usually expressed, and primaries and metastases in other sites, where immunohistochemical TTF-1 staining is normally not observed. Given the high frequency of primary lung carcinomas and metastases, both from extrapulmonary primaries in the lung and from lung adenocarcinomas in other organs, TTF-1 is a widely used marker with diagnostic (and, consequently, clinical) importance.

In routine pathology diagnostics, the two most commonly applied TTF-1 clones are the SPT24 clone and 8G7G1/1. There is some evidence that the SPT24 clone shows a higher sensitivity and less erratic cytoplasmic staining compared to the 8G7G1/1 clone [8].

Despite the former belief that its immunohistochemical expression was highly restricted to cells of adenocarcinomas derived from the lung and thyroid, recent studies have hinted that the expression of TTF-1 is not as specific as was initially thought, and that it can be also found in primaries and metastases from other body sites (albeit to a lesser extent) [9,10,11]. Among malignant tumors, for which expression of TTF-1 has been reported, adenocarcinomas of the gastrointestinal tract deserve a special mention. In these, nuclear staining with TTF-1 has been observed in up to 5% of cases [12]. An awareness of this fact can help pathologists to avoid diagnostic pitfalls.

Another helpful antibody—particularly in the diagnosis of adenocarcinomas of the lungs—is Napsin A. Napsin A is an enzyme that can be assigned to the aspartate proteases, and t is encoded in the NAPSA gene located on chromosome 19 in section q13.3 [13]. Strong expression of Napsin A has been detected in the cytoplasm of type II pneumocytes and intra-alveolar macrophages [14]. Analogously to TTF1-1, Napsin A is used in pathology to diagnose lung tumors, particularly for the differentiation of non-small-cell lung cancer (NSCLC), and can be detected in up to 90% of lung adenocarcinomas. Several clones of anti-Napsin A antibodies are used in pathohistological routine diagnostics, such as the IP64 clone, the TMU-Ad02 and the KCG1.1 clone [15,16]. Regarding the diagnosis of pulmonary adenocarcinomas, there is evidence that TTF-1 reveals a higher sensitivity (94.1%), whereas Napsin A provides a higher specificity (97.8%) [17].

Misinterpretation of rarely seen (but nevertheless occurring) aberrant staining of tissue markers can lead to misinterpretation of clinical findings, with potentially harmful consequences. To further elucidate the frequency of aberrant TTF-1 and Napsin A expression in gastrointestinal cancers, we performed immunohistochemical staining of large collections of esophageal, gastric and colonic adenocarcinomas using anti-TTF-1 antibodies (SPT24 clone) and anti-Napsin A antibodies (IP64 clone) applied on tissue microarrays (TMAs). We also compared the findings with other pathological findings regarding the tumors, including the analysis of so-called gastrointestinal markers CDX2 and SATB2, and CK20.

## 2. Materials and Methods

Buffered formalin-fixed paraffin-embedded tumor tissues from 854 patients with primary resected adenocarcinomas of the gastrointestinal tract placed in next-generation tissue microarrays (TMAs; Grandmaster, 3D Histech, Budapest, Hungary) were investigated [18].

The TMAs were constructed through robot-assisted digital annotation of the slides from the donor blocks for accurate placement of the TMA cores. Two to six cores of 0.6 mm diameter from each tumor tissue were analyzed for each case. Three different cohorts were included: one cohort of 125 patients with esophageal adenocarcinomas, one cohort of 419 patients with gastric adenocarcinomas and one cohort of 310 patients with colorectal adenocarcinomas [19,20,21]. The tumors of the cohorts comprised all pT and pN categories of all entities. More details regarding the cohorts can be found elsewhere [18,19,20,21]. In total, 12 TMAs with 4217 single cores were analyzed.

Immunohistochemical stains were performed on a Leica Bond III stainer (Leica Biosystems, Newcastle, UK). We used anti-TTF-1 (clone SPT24, 1:400, Novocastra/Leica Biosystems; Tris EDTA 40 min at 95 °C) and anti-Napsin A (cone IP64, 1:400, Novocastra/Leica Biosystems; Tris EDTA 30 min at 95 °C) antibodies. Additionally, stains for CK20 (clone Ks20.8, 1:800; Cell Marque/Merck, Darmstadt, Germany; Tris EDTA, 20 min at 95 °C), CDX2 (clone ERP2764Y, 1:400, Cell Marque/Merck, Tris EDTA 30 min at 95 °C) were performed on the same device and stained for SATB2 (clone EP281, ready to use; Cell Marque/Merck, CC1 Tris buffer 36 min at 95 °C) on a Roche Ventana Benchmark Ultra immunostainer (Roche, Vienna, Austria).

Evaluation was performed by two pathologists (P.N., C.G.), with consent diagnosis in the case of discrepant evaluation results. Cases were considered positive if at least 5% of tumor cells showed weak nuclear (TTF-1, CDX2, SATB2) or cytoplasmic (Napsin A, CK20) staining in at least one core of the TMA [12,22]. Descriptive statistics were carried out using IBM SPSS Statistics 28.0 software (Armonk, NY, USA).

## 3. Results

**Figure 1:**
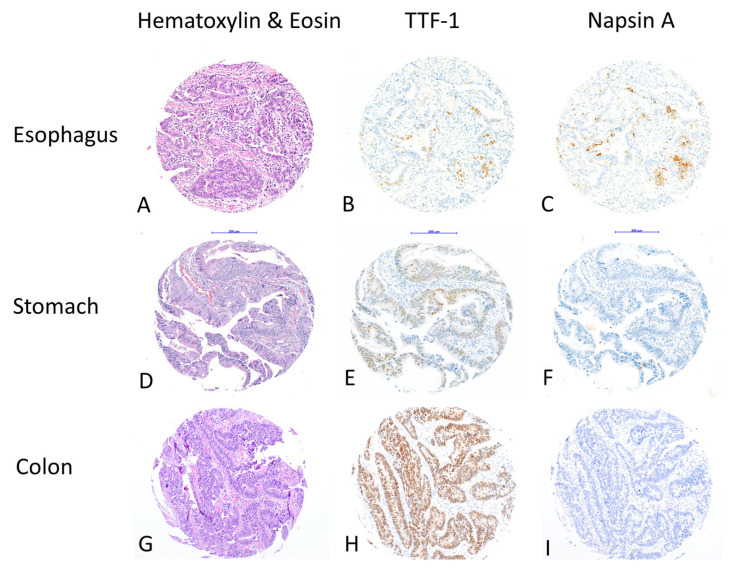
Immunohistochemical expression in gastrointestinal adenocarcinomas with anti-TTF-1 antibodies (SPT24 clone) and anti-Napsin A antibodies (IP64 clone) in tissue microarrays: (**A**–**C**): TTF-1/Napsin A co-expressing esophageal adenocarcinoma: (**A**) hematoxylin–eosin staining; (**B**) TTF-1; (**C**) Napsin A. (**D**–**F**): TTF-1 expressing gastric adenocarcinoma without Napsin A expression: (**D**) hematoxylin–eosin staining; (**E**) TTF-1; (**F**) Napsin A. G-I TTF-1-expressing colon adenocarcinoma without Napsin A expression: (**G**) hematoxylin–eosin staining; (**H**) TTF-1; (**I**) Napsin A. (100× magnification).

In total, 16 cases (16/854; 1.9%) showed positive staining for TTF-1, and 7 cases (7/854; 0.8%) for Napsin A. Five out of seven cases that were Napsin A-positive were also TTF-1-positive. All positive cases had >5% positive tumor cells for both markers (Figure 1).

In detail, five cases (5/125; 4%) of esophageal adenocarcinomas showed positive staining for TTF-1, three cases (3/125; 2.4%) showed positive staining for Napsin A and were both TTF-1- and Napsin A-positive.

Positive TTF-1 staining could be detected in two cases (2/419; 0.5%) of gastric adenocarcinomas and positive Napsin A staining could be detected in in four cases (4/419; 1%) of gastric adenocarcinomas. Two cases (2/419; 0.5%) of gastric adenocarcinomas showed synchronous staining with TTF-1- and Napsin A antibodies.

In colon adenocarcinomas, we observed positive TTF-1 staining in nine cases (9/310; 2.9%), while positive Napsin A staining was not observed in any of these, nor in overall colon cancer cases (Table 1).

Only five tumors showed co-expression of TTF-1 and Napsin A, including three esophageal and two gastric adenocarcinomas. These tumors were of intestinal type according to the Laurén Classification and tubular according the WHO classification, and they were predominantly poorly differentiated. Two gastric cancers showed Napsin A positivity but were TTF-1 negative. Eleven colon adenocarcinomas showed TTF-1 expression but no Napsin A positivity was recorded in these tumors. Additional stainings for so-called gastrointestinal markers revealed CK20 positivity in most cases and CDX2 positivity in some but not all tumors across the locations. Notably, only two of the tumors with dual TTF-1 and Napsin A expression that were exclusively adenocarcinomas of the upper gastrointestinal tract, were CDX2 positive. In contrast, SATB2 expression was only seen in colorectal carcinomas. A detailed pathological description of the tumors with TTF1 and/or Napsin A expression is given in Table 2.

Interestingly, neither TTF-1 nor Napsin A showed a homogenous staining pattern (i.e., the same percentage of positively stained tumor cells) across the TMA cores: the 16 cases that were diagnosed as TTF-1-positive contained 98 cores available for analysis and only 35 (35.7%) showed positive stained tumor cells. The percentage of positive cores per case was highest in gastric adenocarcinomas (3/6 cores; 50%, in two cases), followed by esophageal adenocarcinomas (13/30 cores; 43.3%, in five cases) and colon adenocarcinoma (19/62 cores; 30,6%, in nine cases). The seven cases with Napsin A positivity contained 30 cores, including 15 with positive tumor cells (50%). Similarly to TTF-1, in regards of Napsin A positivity, gastric adenocarcinomas showed the highest percentage (6/12 cores; 50%, in four cases), followed by esophageal adenocarcinomas (9/24 cores; 37,5%, in three cases). A detailed list of the cases with positive stainings for TTF-1 and Napsin A with respect to the single TMA cores is given in Table 3.

**Figure 2:**
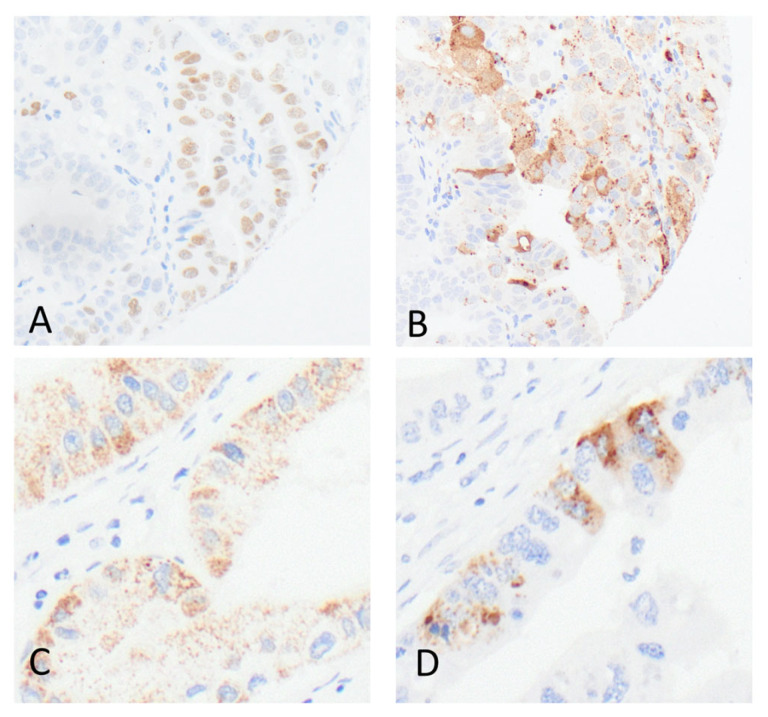
Detailed images of immunohistochemical stainings with anti-TTF-1 antibodies (SPT24 clone) and anti-Napsin A antibodies (IP64 clone). (**A**) Regular nuclear TTF-1 staining in a gastric adenocarcinoma (200× magnification); (**B**) regular cytoplasmatic staining in the same gastric adenocarcinoma (200× magnification); (**C**,**D**) unspecific cytoplasmic TTF-1 staining in two gastric adenocarcinomas (400× magnification).

Unspecific cytoplasmic staining with antibodies against TTF-1 (which is not considered as indicative of TTF-1 positivity) could be observed in 34 (34/4217; 0.8%) cores of 20 cases (20/854; 2.3%), 11 of which were located in the stomach (11/419; 2.6%), and 9 in the esophagus (9/125; 7.2%). None of the colorectal cancer cases showed cytoplasmic staining. Detailed images of the different staining patterns (regular for TTF-1 and Napsin A and unspecific cytoplasmic staining for TTF1) are shown in Figure 2.

## 4. Discussion

TTF-1 IHC and Napsin-A IHC are widely used by pathologists for confirmation of the diagnosis of lung adenocarcinoma, as well as thyroid carcinoma or their metastases and for distinction from other malignant entities [23,24,25,26,27,28]. With the lung being a common site of metastases and histopathological features possibly being insufficient to permit an unequivocal diagnosis, the distinction of primary lung adenocarcinoma from lung metastases of another adenocarcinoma with an extra-pulmonary site can be challenging, is of great importance and has also a deep impact on further therapeutic measures [29,30]. Using the clones SPT24 for TTF-1 and IP64 for Napsin A, we showed that these markers are expressed in a small number of esophageal, gastric and colorectal adenocarcinomas. This is in line with some previous studies, which showed that both TTF-1 and Napsin A are not exclusively expressed in adenocarcinomas of the lung and thyroid gland, but also in other tumor entities (albeit to a lower extent) [11,17,23,24,25].

### 4.1. TTF-1 Expression in Gastrointestinal and Esophageal Adenocarcinomas

We detected TTF-1 positivity in 5 out of 125 cases of esophageal adenocarcinoma (4%), which corresponds to the results of the study by Möller et al., who observed a 3.1% prevalence of TTF-1-positive esophageal adenocarcinomas in their large study on TTF-1 expression in >17,000 tumors of various origins. Here, 3% of 65 esophageal adenocarcinomas were TTF-1-positive, partly with weak staining and partly with strong staining [17]. Of note, Möller et al. used the EP1584Y clone for their studies. Choi et al. reported a frequency of 25% TTF-1 positivity among adenocarcinomas of the gastroesophageal junction, a result that strongly contrasts to ours and to those in the study of Möller et al., which could only partly be explained by the evaluation criteria or staining protocols [17,30]. In Nelson G. Ordonez’s review article, no TTF-1 positivity in esophageal adenocarcinomas was reported; however, IHC was applied in only three cases [25].

In gastric adenocarcinomas, we observed a positive immunohistochemical staining with antibodies against TTF-1 in 2 of 419 cases (0.5%). This very low rate is consistent with the study by Matoso et al., who used the same SPT24 clone and reported one TTF-1 positive case out of 110 (0.9%) [10]. If the 8G7G3/1 clone is applied, the proportion of TTF-1-positive gastric cancers appears to be somewhat higher [25]. In the study of Möller et al., the frequencies of TTF-1-positive gastric cancers were between 0% in gastric adenocarcinomas of diffuse type, and 5.9% in gastric adenocarcinomas of intestinal type, while gastric adenocarcinomas of mixed type revealed a TTF-1 positivity frequency of 5% [17]. This is in line with our observations, where the positive esophageal and gastric cancers were all intestinal-type according to the Laurén classification, and tubular according to the WHO classification. Other investigators have reported remarkably higher incidence rates of TTF-1-positive gastric adenocarcinomas, reaching values as high as 33% in antral gastric adenocarcinomas [30]. In our opinion, however (and also in agreement with other investigators), such high values appear to be somewhat excessive and can probably be explained, to a certain extent, by tumor heterogeneity and the use of a different approach with regard to the analyzed tissue. Whereas we and the majority of investigators have used cores in TMAs, Choi et al. applied immunohistochemical staining on representative sections of paraffin-embedded tissue blocks as well as lymph node metastases, which provide analyzable tumor tissue to a much greater extent [30].

Our result, indicating 2.9% TTF-1-positive cases of colorectal adenocarcinoma, is quite close to the results of other investigators who used the same SPT24 clone, reporting frequencies up to 5.8% [11,17,25]. Lower frequencies of about 1.8% TTF-1-positive cases among colorectal carcinomas have been observed in studies that used the 8G7G3/1 clone, which reportedly appears to have a lower sensitivity than the SPT24 clone [25]. The reason for the aberrant staining of the transcription factor TTF-1 in gastrointestinal tumors, however, has not yet been determined. It has been noted that benign gastric glands can also present TTF-1 positivity, but no further data about this peculiar finding are available [30].

### 4.2. Napsin a Expression in Gastrointestinal and Esophageal Adenocarcinomas

In analogy to TTF-1, Napsin A expression is found not only in adenocarcinomas of the lung, but also in other entities. This includes up to 40% of clear cell carcinomas of the kidney and the ovary, and around 5% of adenocarcinomas of the stomach or extra-pulmonary mucinous adenocarcinomas—particularly those originating from the gastrointestinal tract [13,15,31,32]. Napsin A shows a lower sensitivity (87.4%) than TTF-1, but a significantly higher specificity (97.8%) for lung adenocarcinoma [33].

We detected positive immunohistochemical staining with antibodies against Napsin A (IP64 clone) in 3 out of 125 esophageal adenocarcinomas (2.4%), and 4 out of 419 gastric adenocarcinomas (1%), while no adenocarcinoma of colorectal origin presented with Napsin A positivity. Other investigators have described partly significantly differing data, where Napsin A positivity was seen in up to 83% (five/six) of esophageal adenocarcinomas and in up to 40% (two/five) of gastric adenocarcinomas when polyclonal antibodies were used; however, no positive immunohistochemical reaction was observed in the same tumor cases when a monoclonal anti-Napsin A antibody was applied [34,35]. In analogy to this observation, Mukhopadhyay et al. detected Napsin A positivity in colonic adenocarcinomas only when polyclonal antibodies were used, but no positive reaction in the case of application of monoclonal anti-Napsin A antibodies [34].

### 4.3. Consequences of Aberrant TTF-1 and Napsin a Expression for Pathological and Clinical Diagnostics

The sensitivity of TTF-1 is as high as 94.1%, whereas its specificity of 86% appears to be sub-optimal [17]. Therefore, some authors have suggested a combined analysis using both TTF-1 and Napsin A, reaching a sensitivity of 84.9% and a specificity of 99.1% for the distinction of lung adenocarcinomas from their counterparts in other anatomical sites [33]. This concept is supported by the results of some authors, who failed to show co-expression of TTF-1 and Napsin A in colonic and gastric carcinomas [17]. We did not observe co-expression of TTF-1 and Napsin A in our series of colorectal carcinomas, and only in 2 out of 419 gastric (0.5%) and 3 out of 125 esophageal cancer cases (2.4%), thereby potentially decreasing the rate of tumor misinterpretation when using both antibodies to distinguish them from lung adenocarcinomas. Interestingly, all gastroesophageal adenocarcinomas with co-expression of TTF-1 and Napsin A were of tubular/intestinal type and poorly differentiated. The total low number of cases, however, would not allow to draw any significant conclusions from this observation.

In the light of the fact that the results of several other authors correspond to ours with regard to TTF-1 expression in colonic and gastric adenocarcinomas, some investigators have suggested to resolve the diagnostic dilemma in terms of the distinction of primary lung adenocarcinoma from metastatic colonic carcinomas using “gastrointestinal immunohistochemical markers” [8,9,11,36,37,38,39,40,41,42]. This concept is supported by the observation that a large proportion of TTF-1-positive colonic or gastric cancers also showed an immunohistochemical expression of at least one of the following markers: SATB2, CK20, FABP1, and Villin-1. However, 22% of TTF-1-positive lung adenocarcinomas showed the expression of at least one of the abovementioned “gastrointestinal markers” as well [17]. Others have stated that the frequency of expression of these so-called gastrointestinal markers in lung adenocarcinomas ranges between 3 and 28% [43,44,45]. The application of a combination of markers including IMP3, CDX2, and N-cadherin increased the discriminatory power for the differentiation between esophageal and pulmonary adenocarcinomas, but there were still cases that could not be classified correctly [46]. This may be related to the fact that neither CK20 nor CDX2 are completely specific for intestinal differentiation. Vice versa, enteric type and mucinous lung adenocarcinomas frequently show expression of CDX2 and CK20, often also without TTF-1 and Napsin A expression. In those cases, histopathology, including immunohistochemistry, may not be able to differentiate between a primary lung and gastrointestinal tumor and careful clinical work-up is mandatory [47]. In our case collections of gastrointestinal tumors, most cases with TTF-1 and/or Napsin A expression were CK20-positive and SATB2 expression was restricted to colorectal adenocarcinomas. In the tumors with dual TTF-1 and Napsin A expression, which were all upper gastrointestinal adenocarcinomas, CDX2 expression was seen in some but not all cases, comparable to previous studies [17], demonstrating some usefulness in performing additional immunohistochemical stainings to prove gastrointestinal origin, but still requiring clinical correlation.

### 4.4. Heterogeneity of TTF-1 and Napsin a Staining

Our study provides also data regarding the heterogeneity of TTF-1 and Napsin A expression in gastrointestinal tumors. Only 35 out of 98 scores (35.7%) throughout the TTF-1-positive cases (i.e., TTF-1-positive staining could be detected in at least one core) actually showed positive staining with antibodies against TTF-1, while 15 out of 30 cores (50%) of tumors considered Napsin A-positive of the same origins revealed Napsin A positivity. To the best of our knowledge, such observations have not been explicitly reported in other studies yet; this provides additional hints regarding tumor heterogeneity, which could serve as an explanatory approach to why the results of different studies partly significantly differ from each other. Moreover, heterogeneity may also cause problems in daily routines, particularly when dealing with small tissue samples—not only in the diagnosis of primary tumors, but also of metastases, including cytology specimens [48].

### 4.5. Other Aspects of Aberrant TTF-1 and Napsin a Staining

Notably, neuroendocrine tumors can show TTF-1- and Napsin-A positivity across various primary tumor sites, including the gastrointestinal tract. Including these tumors in studies may have resulted in falsely high positivity rates [49]. Our case collections did not include neuroendocrine tumors, which was assured by careful composition of our investigation cohorts with an initial secondary histological review of all cases after their identification in the pathology databases.

Cytoplasmic staining of TTF-1—particularly if the less specific 8G7G3/1 clone is used—has been suggested to be due to cross-reactivity to mitochondrial proteins, thus probably representing a mitochondrial staining pattern [11,30,50]. Moreover, TTF-1 cytoplasmic staining is observed in normal hepatocytes and hepatocellular carcinomas [51,52]. Despite the first descriptions of this phenomenon, however, more detailed investigations have not been performed so far, leaving nuclear staining as the only pattern for TTF-1 that is considered diagnostic. The reason for the observed aberrant nuclear staining in gastrointestinal and other tumors, however, still remains unclear.

## 5. Conclusions

TTF-1 and Napsin A are valuable immunohistochemical markers for diagnosing pulmonary adenocarcinoma and thyroid carcinoma, helping differentiate them from metastases of other origins. However, this study highlights that a small number of upper gastrointestinal adenocarcinomas (particularly poorly differentiated carcinomas) showed dual TTF-1/Napsin A positivity, while no colorectal cancers exhibited this pattern. Based on our observations we conclude that—provided that the immunohistochemistry protocols work under continuous quality control conditions to ensure the best and most accurate staining results—a particularly patchy or heterogenous staining pattern of TTF-1 and Napsin-A does not mean pulmonary origin. Combining TTF-1 with Napsin A reduces misinterpretation risks in most cases, since Napsin A positivity, which should be detectable in pulmonary adenocarcinomas, was absent in colorectal tumors. In addition, although no definitive gastrointestinal-specific markers exist, additional investigations for CK20, CDX2 and SATB2 may add further information, but thorough clinical correlation for tumors with atypical marker profiles is necessary. Awareness of TTF-1/Napsin A expression in rare extrapulmonary cancers, and their occasional absence in lung adenocarcinomas, is essential to avoid diagnostic errors.

## Figures and Tables

**Table 1:** TTF-1, Napsin A and synchronous immunohistochemical TTF-1 and Napsin A expression in colorectal, gastric and esophageal adenocarcinomas.

Tumor Entity	TTF-1-Positive	Napsin A-Positive	TTF-1/Napsin A Co-Expression
Esophageal adenocarcinoma	5/125 (4%)	3/125 (2.4%)	3/125 (2.4%)
Gastric adenocarcinoma	2/419 (0.5%)	4/419 (1%)	2/419 (0.5%)
Colon adenocarcinoma	9/310 (2.9%)	0/310 (0%)	0/310 (0%)
Total	16/854 (1.9%)	7/854 (0.8%)	5/854 (0.6%)

**Table 2:** Pathological characterization of TTF-1- and Napsin A-expressing tumors.

Case/Sex	Location	WHO/Laurén Type	Tumor Grading	pT and pN Category	CK20	CDX2	SATB2
**Dual TTF-1- and Napsin A-expressing tumors**							
1/male	Esophagus	Tubular/intestinal	G3	pT1 N0	pos	pos	neg
2/male	Esophagus	Tubular/intestinal	G3	pT3 N+	pos	neg	neg
3/male	Esophagus	Tubular/intestinal	G3	pT3 N0	pos	neg	neg
4/male	Stomach	Tubular/intestinal	G2	pT1b N+	pos	pos	neg
5/male	Stomach	Tubular/intestinal	G3	pT1a N0	pos	neg	neg
**TTF-1-expressing, Napsin A-negative tumors**							
6/male	Esophagus	Tubulo-pap./intestinal	G2	pT1b N0	pos	pos	neg
16/male	Esophagus	Tubular/intestinal	G2	pT1a N0	neg	neg	neg
7/male	Colorectum	-	G2	pT3 N0	pos	pos	pos
8/male	Colorectum	-	G3	pT2 N0	pos	pos	pos
9/male	Colorectum	-	G3	pT3 N+	pos	pos	pos
10/male	Colorectum	-	G2	pT3 N+	pos	pos	pos
11/male	Colorectum	-	G2	pT3 N0	pos	pos	pos
12/male	Colorectum	-	G2	pT2 N0	pos	pos	pos
13/male	Colorectum	-	G3	pT3 N0	pos	pos	pos
14/female	Colorectum	-	G3	pT4 N+	neg	neg	pos
15/female	Colorectum	-	G3	pT2 N0	pos	pos	pos
**Napsin A expressing, TTF-1 negative tumors**							
17/male	Stomach	Tubular/intestinal	G2	pT4 N+	pos	pos	neg
18/female	Stomach	Mixed	G3	pT4 N+	pos	neg	neg

**Table 3:** Heterogeneity of staining patterns of TTF-1 and Napsin A in the single TMA cores (cases marked with * are the TTF-1 and Napsin A co-expressing tumors).

Immuno- Histochemistry	Case Number	Location	Total Number of Cores	Number of Positive Cores	Max % of Positive Cells per Core
TTF-1	1 *	esophagus	6	2	20
TTF-1	2 *	esophagus	6	5	40
TTF-1	3 *	esophagus	6	4	30
TTF-1	6	esophagus	6	1	5
TTF-1	16	esophagus	6	1	5
TTF-1	4 *	stomach	3	1	10
TTF-1	5 *	stomach	3	2	10
TTF-1	7	colorectum	6	2	40
TTF-1	8	colorectum	6	1	50
TTF-1	9	colorectum	6	6	100
TTF-1	10	colorectum	6	2	5
TTF-1	11	colorectum	6	2	60
TTF-1	12	colorectum	6	1	20
TTF-1	13	colorectum	6	2	5
TTF-1	14	colorectum	6	3	25
TTF-1	15	colorectum	6	1	5
Napsin A	1 *	esophagus	6	2	15
Napsin A	2 *	esophagus	6	3	40
Napsin A	3 *	esophagus	6	4	100
Napsin A	4 *	stomach	3	2	5
Napsin A	5 *	stomach	3	2	30
Napsin A	17	stomach	3	1	5
Napsin A	18	stomach	3	1	10

## Data Availability

Original data are available from the authors upon request.

## References

[1] Civitareale D., Lonigro R., Sinclair A.J., Di Lauro R. (1989). A thyroid-specific nuclear protein essential for tissue-specific expression of the thyreoglobulin promoter. EMBO J..

[2] Francis-Lang H., Price M., Polycarpou-Schwarz M., Di Lauro R. (1992). Cell-type-specific expression of the rat thyreoperoxidase promotor indicates common mechanisms for thyroid-specific gene expression. Mol. Cell. Biol..

[3] Endo T., Kaneshige M., Nakazato M., Ohmori M., Harii N., Onaya T. (1997). Thyroid-transcription factor-1 activates the promotor activity of rat thyroid Na^+^/I^−^ symporter gene. Mol. Endocrinol..

[4] Bruno M.D., Bohinski R.J., Huelsman K.M., Whitsett J.A., Korfhagen T.R. (1995). Lung cell-specific expression of the murine surfactant protein A (SP-A) gene is mediated by interactions between the SP-A promotor and thyroid transcription factor-1. J. Biol. Chem..

[5] Bohinski R.J., Di Lauro R., Whitsett J.A. (1994). The lung-specific surfactant protein B gene promoter is a target for thyroid transcription factor 1 and hepatocyte nuclear factor 3, indicating common factors for organ-specific gene expression along the foregut axis. Mol. Cell. Biol..

[6] Yan C., Sever Z., Whitsett J.A. (1995). Upstream enhancer activity in the human surfactant protein B gene is mediated by thyroid transcription factor 1. J. Biol. Chem..

[7] Kelly S.E., Bachurski C.J., Burhans M.S., Glasser S.W. (1996). Transcription of the lung-specific surfactant protein C gene is mediated by thyroid transcription factor 1. J. Biol. Chem..

[8] Pan C.C., Chen P.C., Tsay S.H., Chiang H. (2004). Cytoplasmic immunoreactivity for thyroid transcription factor-1 in hepatocellular carcinoma: A comparative immunohistochemical analysis for four commercial antibodies using a tissue array technique. Am. J. Clin. Pathol..

[9] Nakamura N., Miyagi E., Murata S., Kawaoi A., Katoh R. (2002). Expression of thyroid transcription factor-1 in normal and neoplastic lung tissues. Mod. Pathol..

[10] Matoso A., Singh K., Jakob R., Greaves W.O., Tavares R., Noble L., Resnick M.B., Delellis R.A., Wang L.J. (2010). Comparison of thyroid transcription factor-1 expression by 2 monoclonal antibodies in pulmonary and nonpulmonary primary tumors. Appl. Immunohistochem. Mol. Morphol..

[11] Cai Y.-C., Banner B., Glickman J., Odze R.D. (2001). Cytokeratin 7 and 20 and thyroid transcription factor-1 can help distinguish pulmonary from gastrointestinal carcinoid and pancreatic endocrine tumors. Hum. Pathol..

[12] Comperat E., Zhang F., Perrotin C., Molina T., Magdeleinat P., Marmey B., Régnard J.F., Audouin J., Camilleri-Broët S. (2005). Variable sensitivity and specificity of TTF-1 antibodies in lung metastatic adenocarcinoma of colorectal origin. Mod. Pathol..

[13] Weidemann S., Böhle J.L., Contreras H., Luebke A.M., Kluth M., Büscheck F., Hube-Magg C., Höflmayer D., Möller K., Fraune C. (2021). Napsin A Expression in Human Tumors and Normal Tissues. Pathol. Oncol. Res..

[14] Hirano T., Auer G., Maeda M., Hagiwara Y., Okada S., Ohira T., Okuzawa K., Fujioka K., Franzén B., Hibi N. (2000). Human Tissue Distribution of TA02, which is homologous with a new type of aspartic proteinase, Napsin A. Jpn. J. Cancer Res..

[15] Heymann J.J., Hoda R.S., Scognamiglio T. (2014). Polyclonal napsin A expression: A potential diagnostic pitfall in distinguishing primary from metastatic mucinous tumors in the lung. Arch. Pathol. Lab. Med..

[16] Turner B.M., Cagle P.T., Sainz I.M., Fukuoka J., Shen S.S., Jagirdar J. (2012). Napsin A, a new marker for lung adenocarcinoma, is complementary and more sensitive and specific than thyroid transcription factor 1 in the differential diagnosis of primary pulmonary carcinoma: Evaluation of 1674 cases by tissue microarray. Arch. Pathol. Lab. Med..

[17] Möller K., Gulzar T., Lennartz M., Viehweger F., Kluth M., Hube-Magg C., Bernreuther C., Bawahab A.A., Simon R., Clauditz T.S. (2024). TTF-1 is a highly sensitive but not fully specific marker for pulmonary and thyroidal cancer: A tissue microarray study evaluation more than 17,000 tumors from 152 different tumor entities. Virchow Arch..

[18] Zlobec I., Suter G., Perren A., Lugli A. (2014). A Next-generation Tissue Microarray (ngTMA) Protocol for Biomarker Studies. J. Vis. Exp..

[19] Dislich B., Stein A., Seiler C.A., Kröll D., Berezowska S., Zlobec I., Galvan J., Slotta-Huspenina J., Walch A., Langer R. (2017). Expression patterns of programmed death ligand-1 in esophageal adenocarcinomas: Comparison between primary tumors and metastases. Cancer Immunol. Immunother..

[20] Dislich B., Blaser N., Berger M.D., Gloor B., Langer R. (2020). Preservation of Epstein-Barr virus status and mismatch repair protein status along the metastatic course of gastric cancer. Histopathology.

[21] Bauer K., Nitsche U., Slotta-Huspenina J., Drecoll E., von Weyhern C.H., Rosenberg R., Höfer H., Langer R. (2012). High HSP27 and HSP70 expression levels are independent adverse prognostic factors in primary resected colon cancer. Cell. Oncol..

[22] Bae J.M., Kim J.H., Park J.H., Park H.E., Cho N.Y., Kang G.H. (2018). Clinicopathological and molecular implications of aberrant thyroid transcription factor-1 expression in colorectal carcinomas: An immunohistochemical analysis of 1319 cases using three different antibody clones. Histopathology.

[23] Si Kei L., Oyedele A.A. (2020). Practical Application of Lineage-Specific Immunohistochemistry Markers. Arch. Pathol. Lab. Med..

[24] Pegolo E., Machin P., Damante G., Di Loreto C. (2014). TTF-1 Positivity in 2 Cases of Adenocarcinoma of the Gastrointestinal Tract. Appl. Immunohistochem. Mol. Morphol..

[25] Ordonez N.G. (2012). Value of Thyroid Transcription Factor-1 Immunostaining in Tumor Diagnosis: A Review and Update. Appl. Immunihistochem. Mol. Morphol..

[26] Moldvay J., Jackel M., Bogos K., Soltész I., Agócs L., Kovács G., Schaff Z. (2004). The role of TTF-1 in differentiating primary and metastatic lung adenocarcinomas. Pathol. Oncol. Res..

[27] Zhang H., Liu J., Cagle P.T., Allen T.C., Laga A.C., Zander D.S. (2005). Distinction of pulmonary small cell carcinoma from poorly differentiated squamous cell carcinoma: An immunohistochemical approach. Mod. Pathol..

[28] Porcel J.M. (2018). Biomarkers in the diagnosis of pleural diseases: A 2018 update. Ther. Adv. Respir. Dis..

[29] Mangiameli M., Cioffi U., Alloisio M., Testori A. (2022). Lung metastases: Current surgical indications and new perspectives. Front. Surg..

[30] Choi S.M., Furth E.E., Zhang P.J. (2016). Unexpected TTF-1 Positivity in a Subset of Gastric Adenocarcinomas. Appl. Immunohistochem. Mol. Morphol..

[31] Yamashita Y., Nagasaka T., Naiki-Ito A., Sato S., Suzuki S., Toyokuni S., Ito M., Takahashi S. (2015). Napsin A is a specific marker for ovarian clear cell adenocarcinoma. Mod. Pathol..

[32] Theodoropoulos D.S., Ledfort D.K., Lockey R.F. (2000). Expression of the lung-specific thyroid transcription factor (TTF-1) within the tracheoesophageal fistula of embryo rats exposed to Adriamycin. J. Pediatr. Surg..

[33] Brasch F., Ochs M., Kahne T., Guttentag S., Schauer-Vukasinovic V., Derrick M., Johnen G., Kapp N., Muller K.M., Richter J. (2003). Invovment of napsin A in the C- and N-terminal processing of surfactant protein B in type-II pneumocytes of the human lung. J. Biol. Chem..

[34] Mukhopadhyay S., Katzenstein A.-L.A. (2012). Comparison of monoclonal napsin A, polyclonal napsin A, and TTF-1 determining lung origin in metastatic adenocarcinomas. Am. J. Clin. Pathol..

[35] Takanashi Y., Kurachi K., Fujihiro M., Sekihara K., Torii K., Kawase A., Matsubayashi Y., Hayakawa T., Baba S., Sugimura H. (2023). Thyroid transcription factor-1 expression in rectal adenocarcinoma metastatic to the lung. Respir. Med. Case Rep..

[36] Ye J., Findeis-Hosey J.J., Yang Q., McMahon L.A., Yao J.L., Li F., Xu H. (2011). Combination of Napsin A and TTF-1 immunohistochemistry helps in differentiating primary lung adenocarcinoma from metastatic carcinoma in the lung. Appl. Immunhistochem. Mol. Morphol..

[37] Penman D., Downie I., Roberts F. (2006). Positive immunostaining for thyroid transcription factor-1 in primary and metastatic colonic adenocarcinoma: A note of caution. J. Clin. Pathol..

[38] Xu B., Thong N., Tan D., Khoury T. (2010). Expression of thyroid expression factor-1 in colorectal carcinoma. Appl. Immunohistochem. Mol. Morphol..

[39] Bejarano P.A., Baughman R.P., Biddinger P.W., Miller M.A., Fenoglio-Preiser C., al-Kafaji B., Di Lauro R., Whitsett J.A. (1996). Surfactant proteins and thyroid transcription factor-1 in pulmonary and breast carcinomas. Mod. Pathol..

[40] Alabdullah B., Hadji-Ashrafy A. (2022). Identification of the most specific markers to differentiate primary pulmonary carcinoma from metastatic gastrointestinal carcinoma to the lung. Diagn. Pathol..

[41] Su Y.C., Hsu Y.C., Chai C.Y. (2006). Role of TTF-1, CK20, and CK7 immunohistochemistry for diagnosis of primary and secondary lung adenocarcinoma. Kaohsiung J. Med. Sci..

[42] Malmros K., Lindholm A., Vidarsdottir H., Jirström K., Nodin B., Botling J., Mattsson J.S.M., Micke P., Planck M., Jönsson M. (2024). Diagnostic gastrointestinal markers in primary lung cancer and primary metastases. Virchows Arch..

[43] De Michele S., Remotti H.E., Del Portillo A., Lagana S.M., Szabolcs M., Saqi A. (2021). SATB2in neoplasms of lung, pancreatobiliary, and gastrointestinal origins. Am. J. Clin. Pathol..

[44] Moll R., Robine S., Dudouet B., Louvard D. (1987). Villin: A cytoskeletal protein and a differentiation marker expressed in some human adenocarcinomas. Virchows Arch. B Cell Pathol. Incl. Mol. Pathol..

[45] Sun F., Wang P., Zheng Y., Jia W., Liu F., Xiao W., Bao J., Wang S., Lu K. (2018). Diagnosis, clinicopathological characteristics and prognosis f pulmonary mucinous adenocarcinoma. Oncol. Lett..

[46] Aulakh K.S., Chisholm C.D., Smith D.A., Speights V.O. (2013). TTF-1 and napsin A do not differentiate metastatic lung adenocarcinomas from primary esophageal adenocarcinomas: Proposal of a novel staining panel. Arch. Pathol. Lab. Med..

[47] Inamura K., Satoh Y., Okumura S., Nakagawa K., Tsuchiya E., Fukayama M., Ishikawa Y. (2005). Pulmonary adenocarcinomas with enteric differentiation: Histologic and immunohistochemical characteristics compared with metastatic colorectal cancers and usual pulmonary adenocarcinomas. Am. J. Surg. Pathol..

[48] Li W., Cohen M.B. (2021). TTF-1, napsin A and CDX2 co-expression in metastatic rectal adenocarcinoma to the lung. Cytopathology.

[49] Yun J.P., Zhang M.F., Hou J.H., Tian Q.H., Fu J., Liang X.M., Wu Q.L., Rong T.H. (2007). Primary small cell carcinoma of the esophagus: Clinicopathological and immunohistochemical features of 21 cases. BMC Cancer.

[50] Pang Y., von Turkovich M., Wu H., Mitchell J., Mount S., Taatjes D., Cooper K. (2006). The binding of thyroid transcription factor-1 and hepatocyte paraffin 1 to mitochondrial proteins in hepatocytes: A molecular and immunoelectron microscopic study. Am. J. Clin. Pathol..

[51] Lei J.Y., Bourne P.A., diSant’Agnese P.A., Huang J. (2006). Cytoplasmic staining of TTF-1 in the differential diagnosis of hepatocellular carcinoma vs. cholangiocarcinoma and metastatic carcinoma of the liver. Am. J. Clin. Pathol..

[52] Bejarano P.A., Mousavi F. (2003). Incidence and significance of cytoplasmic thyroid transcription factor-1 immunoreactivity. Arch. Pathol. Lab. Med..

